# Exploring the Research Focus of RNA-Binding Proteins in Trauma and Burns

**DOI:** 10.1155/ancp/5587781

**Published:** 2024-12-30

**Authors:** Sujie Xie, Yifan Liu, Wei Zhang, Jianyu Lu, Xirui Tong, Jie Huang, Yushu Zhu, Minjuan Wu, Xinya Guo, Hanlin Sun, Minyi Gu, Luofeng Jiang, Shuyuan Xian, Runzhi Huang, Zhaofan Xia, Shizhao Ji

**Affiliations:** ^1^Department of Burn Surgery, The First Affiliated Hospital of Naval Medical University, People's Republic of China, Research Unit of Key Techniques for Treatment of Burns and Combined Burns and Trauma Injury, Chinese Academy of Medical Sciences, No. 168 Changhai Road, Shanghai 200433, China; ^2^Department of Urology, Xinhua Hospital Affiliated to Shanghai Jiao Tong University School of Medicine, No. 1665 Kongjiang Road, Shanghai 200092, China

**Keywords:** bibliometrics, burns, meta-analysis, RNA-binding proteins, trauma, wound healing

## Abstract

**Background:** Trauma and burns are leading causes of death and significant global health concerns. RNA-binding proteins (RBPs) play a crucial role in post-transcriptional gene regulation, influencing various biological processes of cellular RNAs. This study aims to review the emerging trends and key areas of research on RBPs in the context of trauma and burns.

**Methods:** A series of relevant articles were manually reviewed, and scientific publications on RBPs related to trauma and burns were retrieved from the Web of Science Core Collection (WoSCC) on May 19, 2024. Bibliometric analysis was performed using R-bibliometrix, VOSviewer, and CiteSpace. We adhered to the Preferred Reporting Items for Systematic Reviews and Meta-Analyses (PRISMA) guidelines to conduct the meta-analysis, followed by a comprehensive review of the selected papers.

**Results:** A total of 539 publications were identified from 2000 to 2024. China was the most productive and collaborative country, with Zhang Y being the most prolific author and PLoS One being the leading publication source. Keyword analysis revealed four distinct clusters. The thematic analysis identified eight key topics, including “RNA-binding proteins,” “traumatic brain injury,” and “inflammation.” Four studies involving 5.976 patients were included in the final meta-analysis, which indicated a correlation between RBP expression levels and poor prognosis in traumatic brain injury (TBI).

**Conclusion:** Our findings provide valuable insights into the developing trends and key areas of research on RBPs in trauma and burns. Notably, we identified two primary hotspots: RBPs in the pathophysiological mechanisms of various traumatic injuries and RBPs in the processes of cutaneous wound healing. This rapidly evolving field offers significant reference points for scientific researchers and clinical practitioners.

## 1. Introduction

It was reported that unintentional injuries had been the third or fourth leading cause of death all the way from 2015 to 2020 in the United States [1, 2]. With considerably high morbidity and mortality, burn is an underestimated injury to the skin or other organic tissue which can be caused by heat, cold, friction, radiation, and chemical or electronic sources. Burn injuries, especially severe ones, can trigger immunological and inflammatory responses, along with metabolic abnormalities and distributive shock, all of which can be extremely difficult to manage and will result in multiple organ dysfunction syndrome [3]. According to the World Health Organization (WHO) estimation, burn injuries account for approximately 180,000 deaths annually around the world (https://www.who.int/en/news-room/fact-sheets/detail/burns). Additionally, trauma is a serious injury to the body, often resulting from violence or accidents, which is also one of the most relevant but neglected global health concerns [4]. Data from the Centers for Disease Control and Prevention (CDC) revealed that motor vehicle crush and firearm-related injury have been the top 2 leading causes of death among children and adolescents in the United States all the way from 1999 to 2022, which were responsible for deaths more than all other causes combined [5]. According to injured body parts, trauma can be classified into traumatic brain injury (TBI), maxillofacial trauma, neck trauma, thoracic and dorsal trauma, abdominal and lumbar trauma, pelvis trauma, spine and traumatic spinal cord injury (SCI), and limb trauma. Particularly, TBI is responsible for the majority of trauma deaths and a leading cause of disability in patients with trauma [6, 7]. Moreover, diabetic foot ulcers (DFUs) also account for a great number of trauma events, presenting as a severe public issue [8]. Current treatments for trauma and burn wounds are limited due to a lack of comprehensive understanding of the cellular and molecular mechanisms underlying tissue repair and wound healing [9]. Millions of individuals worldwide suffer from poor wound healing as a result of poorly controlled aspects of the healthy tissue repair response, such as inflammation, angiogenesis, matrix deposition, and cell recruitment, the failure of which is usually associated with latent healing disorders, such as diabetes, aging, and vascular disease [10, 11].

RNA-binding proteins (RBPs) are proteins that play an imperative role in post-transcriptional gene regulation (PTGR). RBPs regulate alternative splicing, polyadenylation, maturation, stability, transport, translation, and degradation of cellular coding and noncoding RNAs by directly binding to them or by functioning as integral parts of macromolecular protein complexes that bind to them, hence supervising the formation and function of transcripts and maintaining cell homeostasis [12, 13]. RBP has a highly dynamic spatiotemporal regulation process and important biological functions such as cell transport, localization, development, differentiation, and metabolism [14]. And the abnormal alteration of RBPs will affect the RNA life cycle and produce aberrant protein phenotypes, which can result in the occurrence and development of multiple diseases, including various cancers, trauma, and burns [15–17]. Owing to the rapid development of high-throughput sequencing technologies, the critical roles epigenetic regulation such as DNA methylation, histone modification, and noncoding RNA regulation play in the pathophysiological mechanisms of trauma and burns have been widely reported in this century [18–24]. However, RBPs in trauma and burns have not been systematically analyzed or reviewed yet, which provides us with a promising research field to explore.

Consequently, to start with, we manually read a variety of papers concerned with RBPs in trauma and burns [25–32], and it appeared to us that RBPs in the pathophysiological mechanisms of various traumatic injuries and processes of cutaneous wound healing might be the research hotspots. To obtain more objective results for a comprehensive review of this research area, we employed bibliometric analysis and meta-analysis. Bibliometrics is an auxiliary technique exploiting statistical and visualization tool to conduct a quantitative analysis in a specific research field. And recently, bibliometrics has been widely utilized to illustrate the knowledge structures, developmental trends, and research hotspots of various medical research topics, for instance, intestinal microbiota in rheumatic diseases and trauma [33, 34], fibroblasts in rheumatoid diseases and DFUs [35, 36], gene expression in SCI [37], and sequencing technology in rheumatism [38]. Meta-analysis refers to the statistical process of analyzing and combining results from multiple similar studies [39]. Nevertheless, bibliometric analysis and meta-analysis on RBPs in trauma and burns remain a void. In this study, we employed bibliometric analysis and meta-analysis to conduct an objective examination of papers related to RBPs in trauma and burns. The results were integrated with our manual review of relevant papers to comprehensively elucidate the knowledge structures, developmental trends, and research hotspots in this field.

## 2. Materials and Methods

### 2.1. Data Source and Search Strategy

To identify the global status and trends of scientific publications of RBPs in trauma and burns till 2024, we utilized the Web of Science Core Collection (WoSCC) as our data source for a comprehensive search, which was performed on a single day (May 19, 2024) to reduce database update deviations. Only the WoSCC database has complete citation information to support our bibliometric analysis. Our retrieval strategy was (((TS = “Trauma”) OR (TS = “Traumatic”) OR (TS = “Burn”) OR (TS = “Burns”) OR (TS = “Burning”) OR (TS = “Burn injury”) OR (TS = “Burn injuries”) OR (TS = “Empyrosis”) OR (TS = “Ambustion”) OR (TS = “Scald”) OR (TS = “Scalds”) OR (TS = “Scalding”) OR (TS = “Scalded”) OR (TS = “Wound Healing”)) AND ((TS = “RNA Binding Proteins”) OR (TS = “RNA Binding Protein”) OR (TS = “RNA binding motif”) OR (TS = “RNA binding domain”) OR (TS = “RNA modifier”) OR (TS = “Splicing factor”))). Additionally, among the various literature categories, only articles and reviews were included. Furthermore, the retrieved results were exported into a dataset containing bibliographical information, including affiliations, editors, abstracts, keywords, cited references, and funding details, along with citation information such as authors, document title, document type, year of publication, source, source title, volume, issue, pages, citation count, and digital object identifier. For further analysis, all downloaded records were subsequently integrated into statistical and visualization tools using bibliometric methods.

### 2.2. Statistical Analysis

**Figure 1:**
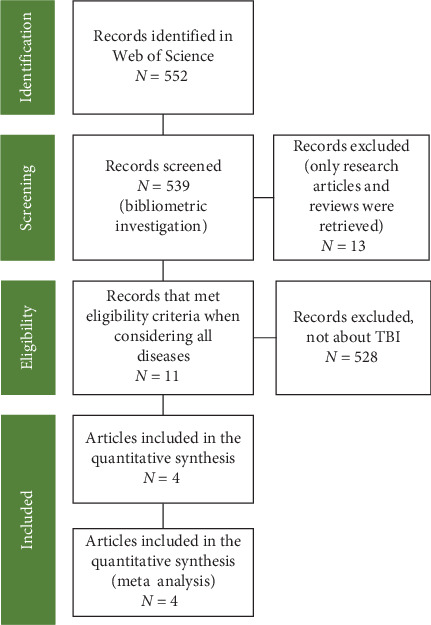
Analytic process of RBPs in trauma and burn research. RBPs, RNA-binding proteins; TBI, traumatic brain injury.

The Preferred Reporting Items for Systematic Reviews and Meta-Analyses (PRISMA) guideline was used [40] to conduct the bibliometric study and meta-analysis presented here, and our procedures adhered to this guideline unless otherwise noted. The flowchart of the process of this meta-analysis was presented in Figure 1. Our study identified a total of 552 articles containing relevant information for this analysis. While conducting our search on the Web of Science, we initially specified the search timeframe to encompass the entire accessible duration from 1637 to 2024. Nevertheless, our citation report (Supporting Information) indicated that the earliest pertinent publication was published in 2000. Upon thorough examination of our search method, we have verified its precision. To ensure clarity and eliminate any potential confusion, we specified the timeframe as 2000–2024 in the text, as all the data depicted in the graphics begin in 2000.

By retaining only those categorized as articles and reviews, we included 539 papers in the bibliometric study. Given that the majority of research on RBPs focuses on fundamental rather than clinical aspects, we conducted a thorough examination of titles and abstracts. As a result, we eliminated 528 papers for various reasons, such as their nonclinical nature or the failure of participants to match the specified inclusion criteria. Subsequently, we evaluated 11 complete articles to determine their suitability and eliminated 7 from further consideration. Ultimately, we incorporated four research including a collective total of 5.976 people.

### 2.3. Bibliometric Study

#### 2.3.1. Inclusion Criteria

The study's inclusion criteria were narrowed down to prioritize data-driven studies and guarantee their relevance. More precisely, articles were considered if they satisfied the following criteria: (a) relevant material on WoSCC up to May 19, 2024, and (b) language requirement, English. As a result of this improvement, 552 pertinent documents were identified. The data obtained from these publications were stored in TXT formats for subsequent studies (Supporting Information).

#### 2.3.2. Exclusion Criteria

Articles not of the review or article type will be excluded.

#### 2.3.3. Bibliometric Analysis

We mainly utilized the Bibliometrix package version 3.2.1 with R version 4.2.2 (Institute for Statistics and Mathematics, Vienna, Austria; www.r-project.org) to perform bibliometric analysis [41]. Additionally, we applied VOSviewer v.1.6.15.0 (Centre for Science and Technology Studies, Leiden University, Leiden, the Netherlands) and CiteSpace (5.8.R3), a visual knowledge graph bibliometric tool based on the Java language for supplementary analysis and validation of results [42, 43]. After extracting bibliographical information and citation information of WoSCC documents in TXT format, we import them in R-bibliometrix analysis, in which we successively conducted overview analysis to require main information table, annual scientific production plot and table, average citations per year plot and table, and three-field plot; source analysis to obtain plots and tables of most relevant sources, most local cited (LC) sources, Bradford's law [44], source impact, and source dynamics; countries and affiliations analysis to retain plots and tables of corresponding author's country, country scientific production, most cited countries, and most relevant affiliations; authors analysis to retrieve plots and tables of most relevant authors, most LC authors, authors production over time, Lotka's law [45], and author impact by *H*-index; document analysis to extract plots and tables of most relevant documents and most LC documents; references analysis to draw plots and tables of most LC references and reference spectroscopy; word analysis to show plots and tables of most frequent words, wordcloud, treemap, word dynamics, and trend topics; clustering analysis to demonstrate clustering of keywords in coupling map; conceptual structure analysis reveal co-occurrence network, thematic map and network, thematic evolution map, plus word map and topic dendrogram in factorial analysis by Multiple Correspondence Analysis (MCA) method; intellectual structure analysis to exhibit cocitation network and histograph; and social structure analysis to illustrate collaboration network and worldmap. In particular, the *H*-index, defined as the number of papers with a citation number of no less than *H*, is exploited to quantify an author's scientific research output and evaluate the impact of his citations [46].

#### 2.3.4. Altmetric Analysis

In addition to conventional bibliometric analysis, we conducted a supplementary investigation into the public's interest in the selected literature items. The Altmetric Attention Score (AAS) serves as a valuable instrument for evaluating social attention, encompassing factors such as visibility on popular social networks like Twitter and Facebook, mentions on scholarly blogs, bookmarks on reference managers like Mendeley, and appearances in news outlets [47]. AAS provides a comprehensive assessment of article attention and measures the dissemination of each publication, rendering it a potential indicator of influence and impact.

The results of publication analysis and reference analysis, especially the historical citation network and cocitation network, along with the results from keyword analysis and thematic analysis were then integrated, based on which we manually got access to hundreds of relevant literature to conclude and review the global status, developmental trends, and research hotspots of RBPs in trauma and burn research.

## 3. Meta-Analysis

### 3.1. Eligibility Criteria

Participants were considered eligible for inclusion if they satisfied the following criteria:

a. Study participants who met the criteria for eligibility had a confirmed diagnosis of TBI that occurred within 24 h of the injury and had their blood tested to detect RBPs within 24 h of the injury.
b. Participants who met the criteria had a Glasgow score documented at the time of the initial occurrence.
c. TBI is a type of brain injury that occurs as a result of an external force. TBI can result from a vigorous impact, collision, or strike to the head or body or from the penetration of an object into the brain.
d. RBPs are proteins that control several processes in the cell involving RNA, such as alternative splicing, polyadenylation, maturation, stability, transport, translation, and destruction of both coding and noncoding RNAs.

### 3.2. Exclusion Criteria

Studies were excluded if they satisfied the following criteria:

a. Patients who had any significant prior neurological condition were not included.
b. The publication types included letters, case reports, meta-analysis, and comments.
c. They possessed inadequate or redundant data. Insufficient data refer to a lack of necessary information, such as incomplete demographic data or an inadequate sample size and number of events. Duplicate data are characterized by the presence of repeated occurrences of identical or highly similar information in a dataset or database, resulting in avoidable redundancy.
d. The research was conducted in languages other than English.

### 3.3. Data Collection and Analysis

Two researchers independently verified and analyzed the literature data, resolving any inconsistencies through negotiation or communication with a third researcher. After gathering high-quality literature, we carry out a series of meta-analyses using Rev Man 5.3 software and STATA15. Relevant information from each paper was extracted and recorded in a spreadsheet: authors' names, year and country of publication, number of patients, number of events (TBI), clinical–epidemiological characteristics of the population, the type of biological sample, the reference or standard gold test, and the index test. We examined sensitivity, specificity, and area under the ROC curve (AUC) as predicting performance indicators. Finally, we evaluated the risk of bias using the QUADAS-C instrument. The QUADAS-C tool was created as a supplement to QUADAS-2 in order to evaluate the potential for bias in comparative studies of diagnostic test accuracy [48].

## 4. Results

### 4.1. Global Scientific Production Analysis

**Figure 2:**
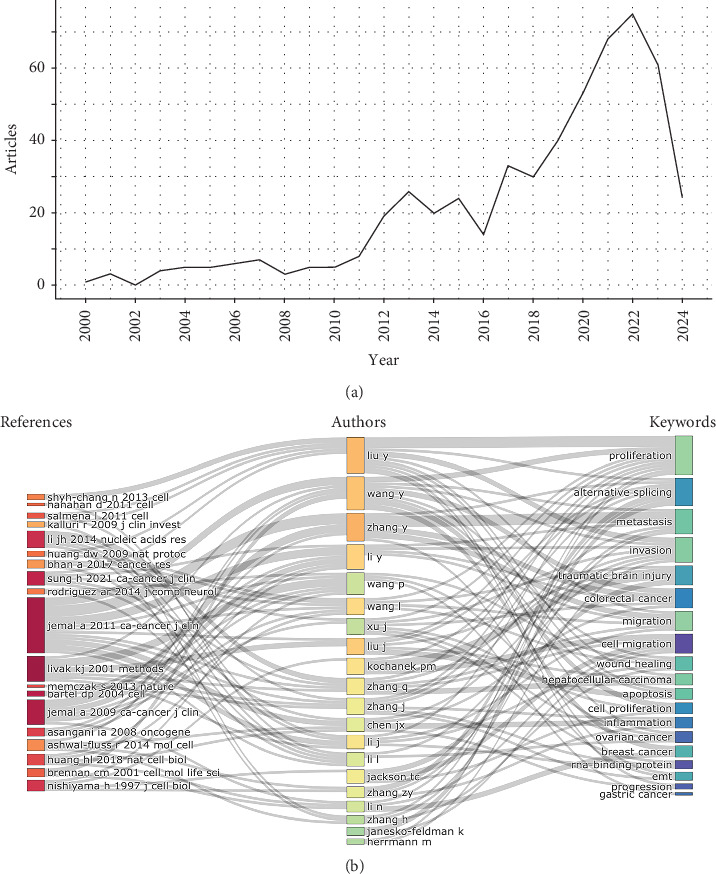
Annual publication volume and key messages. (A) Annual number of publications of RNA-binding proteins (RBPs) in trauma and burn research globally from 2000 to 2024. (B) Three-field plot of RBPs in trauma and burn research. Left field, middle field, and right field represents key references, key authors, and keywords, respectively.

The annual number of articles for RBPs in trauma and burns from 2000 to 2024 were displayed in Figure 2A. With a 15.02% annual growth rate, there were wave-like fluctuations in the variation trend of annual publications, in which a peak emerged recently in 2022 (77 publications, 16.04%). In addition, a three-field plot demonstrated a series of key references cited by various key authors with multiple keywords in Figure 2B.

### 4.2. Country/Region and Affiliation Analyses

**Figure 3:**
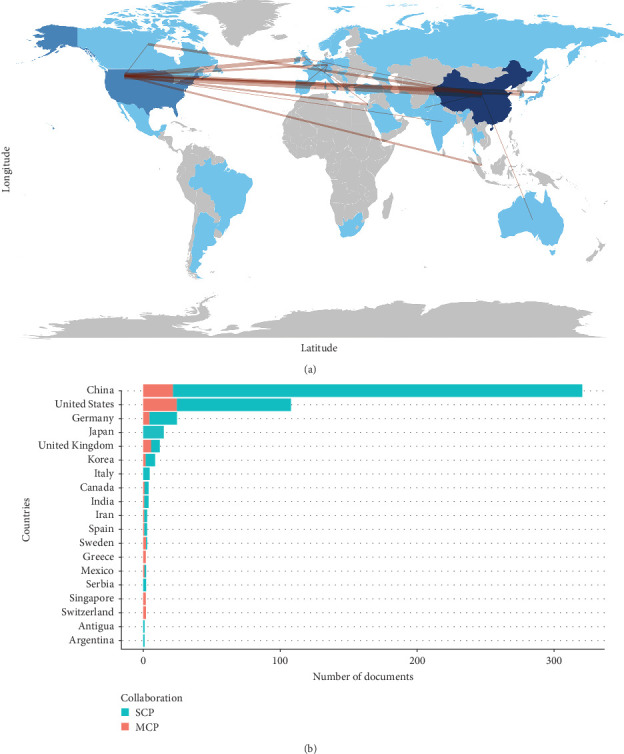
Production of countries/regions. (A) Country/region production and collaboration world map for RBPs in trauma and burn research. The blue color intensity is proportional to the number of publications. The red lines between countries/regions refer to collaborations between them, and the thickness of lines represents the strength of international collaborations. (B) Top 10 country/region production and collaboration histogram for RBPs in trauma and burn research. MCP, multiple-country publications; RBPs, RNA-binding proteins; SCP, single-country publications.

The top 10 most productive countries/regions for RBPs in trauma and burn research were demonstrated in Table 1. Overall, a total of 28 countries/regions participated in this scientific research field, in which the top five most productive countries/regions were China (publications = 321, 59.6%), the United States (publications = 108, 20.0%), Germany (publications = 25, 4.6%), Japan (publications = 15, 2.8%), and the United Kingdom (publications = 12, 2.2%). Furthermore, the country/region production and collaboration world map and network were illustrated in Figure 3A, which revealed that China and the United States were the collaborative centers of all countries/regions. Specifically, the number of single-country publication (SCP) and multiple-country publication (MCP) of the top 10 most productive countries/regions was exhibited in Table 1 and visualized in a histogram in Figure 3B. China (MCP = 22, 6.9%) and the United States (MCP = 25, 23.10%) showed similar results.

### 4.3. Author Analysis

**Figure 4:**
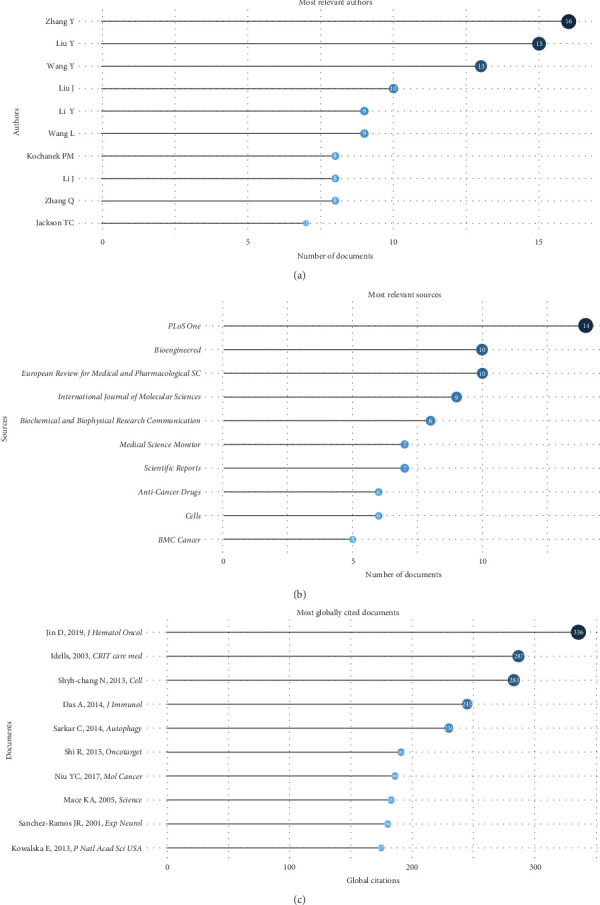
Author, journal, publications, and reference analysis. (A) Top 10 most productive authors for RNA-binding proteins (RBPs) in trauma and burn research. (B) Top 10 most productive journals for RBPs in trauma and burn research. (C) Top 10 most global cited documents for RBPs in trauma and burn research.

The top 10 most productive authors for RBPs in trauma and burn research were displayed in Figure 4A. Besides, the top 10 most LC authors, the top 10 authors with the most impact measured by the *H*-index, and the top 15 most productive journals' production growth over time were, respectively, displayed in Figure S1A–C. In total, the top 10 most productive authors submitted 103 publications, accounting for 19.1% of all articles. Among them, author Zhang Y had the most publications of 16, while Jackson TC had the highest number of LCs of 15, and Zhang Y had the highest *H*-index of 10. They all contributed a lot to the development of the research field.

### 4.4. Journal Analysis

The top 10 most productive journals for RBPs in trauma and burn research were demonstrated in Figure 4B. Moreover, the top 10 most LC journals and the top 10 journals with the most impact measured by the *H*-index were, respectively, shown in Figure S2A,B. In all, the top 10 most productive journals accounted for 82 publications, which was equivalent to 15.2% of the total. Out of all the publications, Public Library of Science (PLoS) One had the largest number of 14, the highest H-index of 10, and LCs of 388. Furthermore, the J*ournal of Biological Chemistry* exhibited the highest LCs, with a value of 710. Each of them made significant contributions to this field of inquiry.

### 4.5. Publication and Reference Analyses

Figure 4C shows the top 10 most cited RBP articles in trauma and burn research global that we analyzed using bibliometric study, and Figure S3A shows the top 10 most cited articles locally. There were 287 TCs and 13.05 TCs per year but 0 LCs for the article “Coagulation, fibrinolysis, and fibrin deposition in acute lung injury” by Idell S. Moreover, the article “Lin28 Enhances Tissue Repair by Reprogramming Cellular Metabolism” by Shyh-Chang N [49] had 9 LCs and 283 GCs, with LCs/GCs of 3.18%. Furthermore, the reference publication year spectroscopy was displayed in Figure S3B. These key studies pioneered the RBPs in trauma and burn research and presented a number of essential methodologies and research models that laid the foundation for the development of the field. What is more, the historical direct citation network and cocitation network were illustrated in Figure S3C, which exhibited the citation relationships among a series of key publications, mostly based on which we reviewed the trends of RBPs in trauma and burn research in the Discussion.

We utilized the Altmetric system to examine the media attention received by top 10 GC and LC publications for RBPs in trauma and burn research. AAS are based on the quality and quantity of mentions in media sources not considered by traditional bibliometrics, and by calculation, each score is assigned a specific weight [50]. While traditional bibliometrics may take time to progress, AAS tracks the immediate dissemination of scholarly works and assesses dissemination shortly after publication [51]. Among them, the article titled “Lin28 enhanced Tissue Repair by Reprogramming Cellular Metabolism” exhibited the highest AAS score.” This study achieved an impressive AAS of 283, and the ranking and number of research outputs are presented in Figure S4A, which were calculated as of January 2, 2024. The global distribution of the 77 users who shared this study is illustrated in Figure S4B, along with the worldwide distribution of its 666 readers (Figure S4C). Notably, a majority of publishers were situated in North America, while readership spanned across diverse regions globally, underscoring the extensive impact exerted by this seminal investigation.

### 4.6. Keyword Analysis

**Figure 5:**
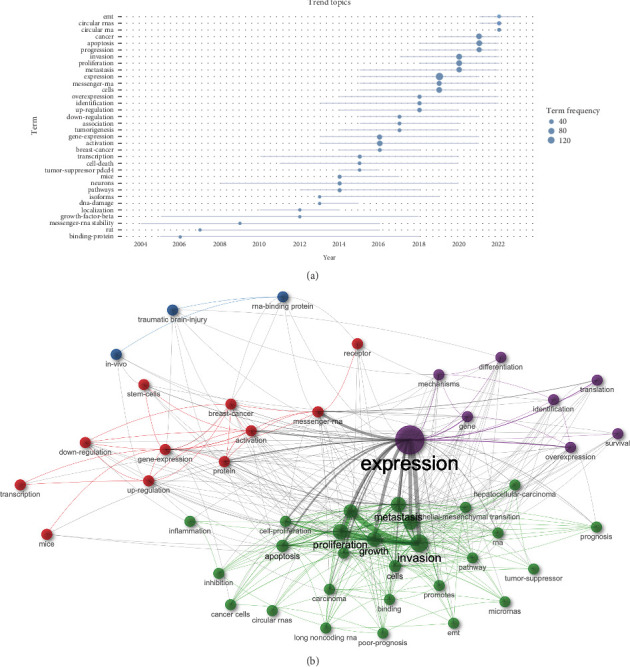
Keyword analysis. (A) Trend topics analysis showing the occurrence trend of the keywords for RNA-binding proteins (RBPs) in trauma and burn research. (B) Keyword co-occurrence network for RBPs in trauma and burn research.

The word cloud (Figure S5A) and tree map (Figure S5B) visually depicted the 50 most commonly used terms in research on RBPs in trauma and burns. The five most often occurring keywords were “expression” (159 occurrences), “invasion” (67 occurrences), “cancer” (60 occurrences), “proliferation” (58 occurrences), and “metastasis” (57 occurrences). In addition, the examination of trending themes revealed the pattern of keyword occurrences for RBPs in trauma and burn research, as shown in Figure 5A. This analysis indicated that “EMT” and “circular RNAs” were highly discussed topics in the past 2 years. More importantly, the keyword co-occurrence network was depicted in Figure 5B, which categorized keywords into four clusters as follows:

  Cluster 1 (purple): the top keywords were “expression,” “gene,” “mechanisms,” “overexpression metastasis,” and “identification.”
  Cluster 2 (green): the top keywords were “invasion,” “metastasis,” “proliferation,” and “growth.”
  Cluster 3 (blue): the top keywords were “RNA-binding protein,” “traumatic brain-injury,” and “in-vivo.”
  Cluster 4 (red): the top keywords were “messenger-RNA,” “activation,” “gene-expression,” and “protein.”

### 4.7. Thematic Analysis

**Figure 6:**
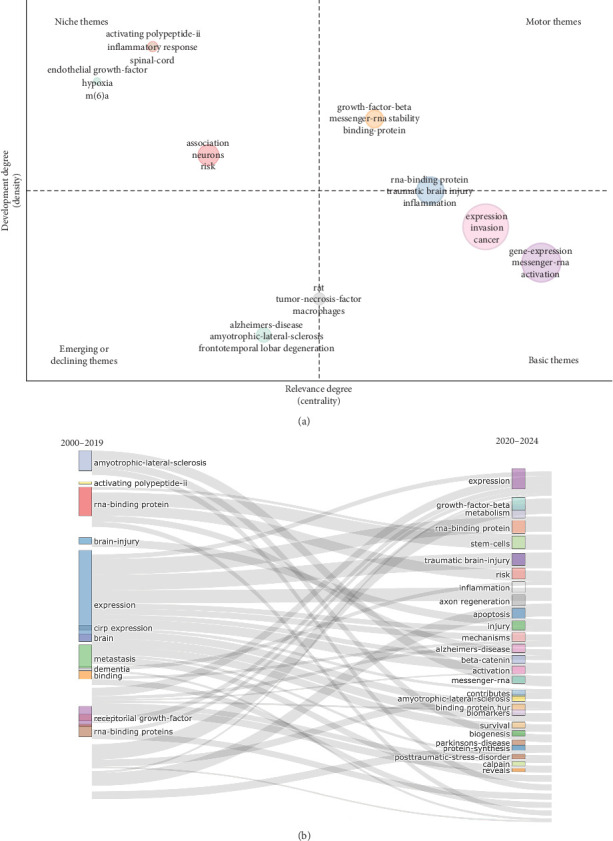
Thematic analysis. (A) Thematic map demonstrates four quadrants of themes as motor themes, niche themes, emerging or declining themes, and basic themes for RNA-binding proteins (RBPs) in trauma and burn research. The horizontal axis refers to relevance degree (centrality), and the vertical axis stands for development degree (density). (B) Thematic evolution from 2000 to 2024 with the cutting point of year 2019 for RBPs in trauma and burn research.

A thematic map demonstrated four quadrants of themes for RBPs in trauma and burn research in Figure 6A. The upper right quadrant represents motor themes, namely, the orange cluster. Within this cluster, the terms “growth-factor-beta,” “messenger-RNA stability,” and “binding-protein” exhibit both high density and high centrality. This indicates that these themes are not only significant but also well-established. Furthermore, a node of fundamental themes was identified in the lower right quadrant, consisting of two clusters: a pink cluster and a purple cluster. These clusters demonstrate their significant relevance but lack substantial progress in this particular area. In addition, the lower left quadrant, which was characterized by rising or declining themes with low density and centrality, contained one node representing “Alzheimers-disease,” “amyotrophic-lateral-sclerosis,” and “frontotemporal lobar degeneration.” This node had limited development and impact in the field. Lastly, in the upper left quadrant, there were three notable themes identified as niche themes: “activating polypeptide-ii,” “inflammatory response,” and “spinal-cord.” These themes were represented as a node. Additionally, the themes “endothelial growth-factor,” “hypoxia,” and “m (6)a” as well as “association”, “neurons”, and “risk” were also identified. These themes, characterized by high density and low centrality, suggest that they are highly developed but isolated from the rest of the field. Apart from those, thematic evolution with the cutting point of year 2019 was shown in Figure 6B, which demonstrated the evolution of top topics from 2000 to 2024 in this research field.

### 4.8. Baseline Characteristics of Included Studies

Due to the limited number of clinical studies on RBP and trauma, only four clinical studies on RBP and TBI were identified throughout the literature review [52–55]. This study comprised five observational studies undertaken in the United States, Europe, and Israel. All trials included both males and females. Furthermore, the data gathering period spanned from 2010 to 2018. The main findings focused on the emergence of new TBI cases. The characteristics of the included studies were summarized in Table 2.

### 4.9. Quality Assessment

In general, the studies had a moderate risk of bias according to the QUADAS-2 [56] (Table 3). All studies were at high or unclear risk of bias in the domain flow and timing. Regarding applicability concerns, Alicia et al. has the same problem. Four studies assessed more than one index test, for which the QUADAS-C tool was used [48]. The risk of bias investigated using QUADAS-C could be interpreted as moderate to poor.

### 4.10. RBPs and TBI

**Figure 7:**
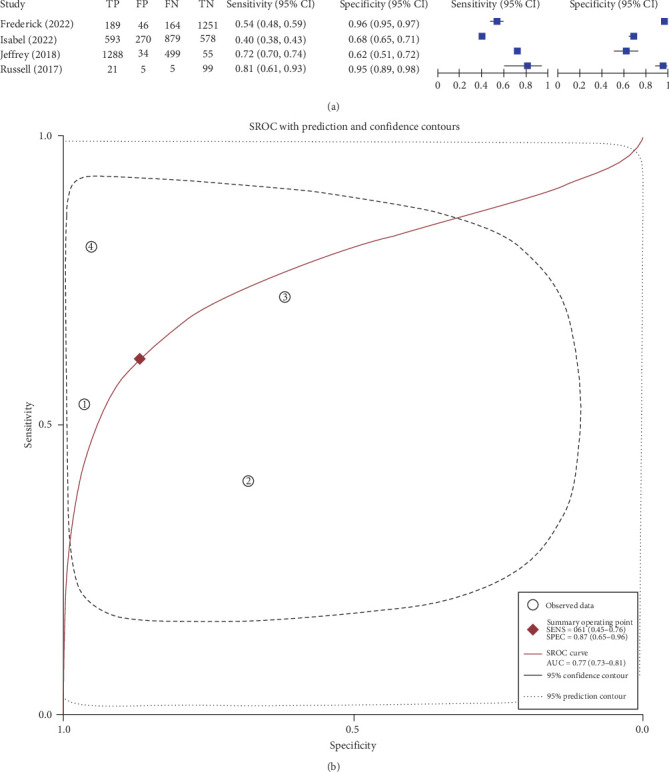
Forest plot of the sensitivity and specificity (A) and the area under the ROC (B) of RNA-binding proteins (RBPs) for predicting unfavorable outcome of traumatic brain injury (TBI).

Four studies examined the impact of RBPs on the prognosis of patients with TBI. The sensitivity was 0.61 (95% CI: 0.45–0.76), and the specificity was 0.87 (95% CI: 0.65–0.96). The AUC was 0.77 (Figure 7A,B). The above results suggest a correlation between RBPs and TBI prognosis. However, due to the presence of numerous proteins within the RBP family, each with distinct functions, a protein-specific analysis is necessary. Consequently, the heterogeneity of this meta-analysis is substantial, and it does not clearly indicate which specific protein increases are positively correlated with poor TBI prognosis. In the Discussion, we will provide a detailed analysis based on the classification of bibliometrics.

## 5. Discussion

**Figure 8:**
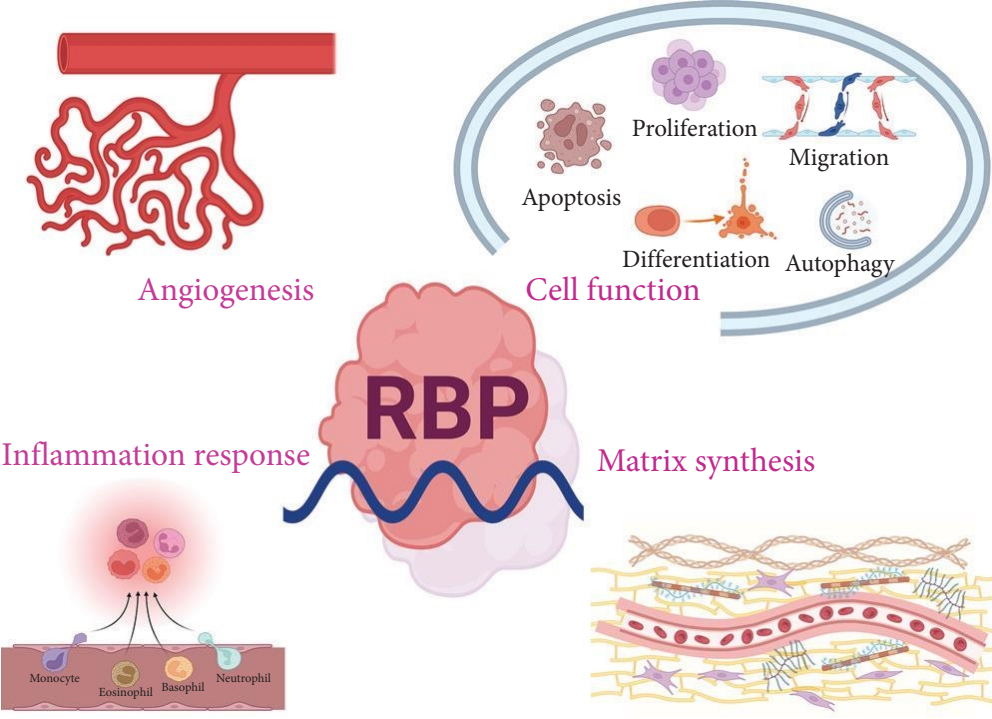
Schematic diagram. RNA-binding proteins (RBPs) play an important role in trauma and burns by influencing angiogenesis, cell function, inflammation response, and matrix synthesis. Created in BioRender.

Trauma and burns are both underappreciated injuries associated with substantial morbidity and mortality, especially among children and adolescents [1, 5]. PTGR is an indispensable process to sustain cellular metabolism, as it coordinates alternative splicing, polyadenylation, maturation, transport, localization, stability, translation, and degradation of all sorts of RNAs, which were regulated by the formation of different ribonucleoprotein (RNP) complexes with RBPs at their core mechanistically [12]. After manually reading a variety of papers concerned with RBPs in trauma and burns [25–32], we considered that researchers focus on RBPs in the pathophysiological mechanisms of various traumatic injuries and processes of cutaneous wound healing by influencing angiogenesis, cell function, inflammation response, and matrix synthesis (Figure 8). So in the present study, to obtain more objective results as references for a comprehensive review of this research field, we performed a comprehensive bibliometric analysis to explore the developmental trends and hotspots of scientific research on RBPs in trauma and burns from the WoSCC employing the Bibliometrix package in R, VOSviewer, and CiteSpace. From that, we obtained 539 publications which were written by 3170 authors from 2000 to 2024. The annual number of publications and cited frequencies have both been on the rise these years, which suggests that RBPs in trauma and burns are arousing increasing attention and have predominant development potential and clinical implication. In author analysis, we identified a bunch of authors such as Liu Y, Li Y, Kochanek PM, and Zhang Y who had contributed remarkably to this research field. Additionally in journal analysis, it was revealed that a series of core journals including *PLoS One*, *Autophagy*, and the *Journal of Biological Chemistry* participated actively and had substantial influence in this research field. Our review and meta-analysis showed that the change of RBP was associated with the severity of TBI.

Further, we integrated the results of publication analysis and reference analysis. The keyword co-occurrence network categorized keywords into four clusters, which could be summarized two hotspots as RBPs in the pathophysiological mechanisms of various traumatic injuries and RBPs in the pathophysiological processes of cutaneous wound healing. This field is rapidly developing and our study provides a valuable reference for scientific researchers and clinical practitioners.

### 5.1. RBPs in the Pathophysiological Processes of Cutaneous Wound Healing

#### 5.1.1. Cluster 1 (Purple) Has the Highest Occurrence of Keywords Such as “Expression,” “Gene,” “Mechanisms,” “Overexpression Metastasis,” and “Identification”

Cutaneous wounds can be caused by a plethora of injuries such as traumatic ones, burns, and diabetes mellitus. Besides, the wound healing process is quite dynamic, interactive, and intricate, involving multiple cells and various proteins including RBPs [57, 58]. Particularly, poor wound healing, especially in chronic or nonhealing wounds, is an intractable issue which commonly occurs in patients with diabetes mellitus, wound infections, and venous or pressure ulcers, which can contribute to serious infections and even limb amputations, as well as increased risk of cardiovascular events [30].

TATA-Box-binding protein-associated factor 15 (TAF15) was revealed to be recruited by exosomal circRNA-itchy E3 ubiquitin protein ligase from bone marrow stromal cells to bind and stabilize the mRNA of nuclear factor erythroid 2-related factor 2 (Nrf2), which would subsequently activate the Nrf2 signaling pathway to facilitate wound healing of DFU [59]. Besides, YTH domain containing 1 (YTHDC1) was also explored in 2022 to modulate the nuclear mRNA stability of sequestosome 1 (SQSTM1), which promoted the autophagy of keratinocytes and facilitated diabetic wound healing [60]. Moreover, another RBP HuR was also able to interact and cooperate with YTHDC1 to regulate SQSTM1 expression. More interestingly, HuR was well investigated to play a role in keratinocyte migration and promoted wound healing of leg ulcers earlier in 2012 [61].

What is more, cold-inducible RNA-binding protein (CIRP) was also explored in wound healing by Idrovo et al. [62] and Higashitsuji et al. [63] in two different studies; however, they came out with distinct conclusions. On the one hand, it was demonstrated that the inflammation phase was facilitated, and wound healing was promoted by CIRP deficiency. On the other hand, CIRP was revealed to accelerate wound healing by promoting cell migration via AMP-activated protein kinase.

#### 5.1.2. Cluster 2 (Green) Was Characterized by the Prominent Phrases “Invasion,” “Metastasis,” “Proliferation,” and “Growth”

It was reported by Sun et al. [64] that Acheron/LARP6 (La ribonucleoprotein domain family, member 6) could interact with Id1 and B1 integrin, promoting the vascular endothelial growth factor (VEGF) mRNA expression, which subsequently improved endothelial proliferation, angiogenesis, and wound healing. In addition, an interesting study investigates that during *Candida albicans* infection, deregulated AU-rich element-binding factor 1 (AUF1) triggered BMP-EZH2-mediated delayed wound healing [65]. Furthermore, RBP muscleblind-like1 (MBNL1) was characterized to regulate the differentiation of myofibroblasts and remodeling of fibrosis, thus accelerating wound healing process [66].

Specifically in corneal wound healing, RNA methyltransferase NSUN 2 was involved in m5C modification of ubiquitin-like containing PHD and RING finger domains 1 (UHRF1) mRNA, facilitating wound healing of corneal epithelium [28]. Moreover, a highly conserved RBP, Lin-28 homolog A (LIN28A), was early identified to augment tissue repair by reprogramming cellular metabolism in 2013 [49]. Lin28A was then shown to play a part in wound healing process of the injured dentin–dental pulp complex [67]. Last but not the least, research on RBPs in burn wound healing, nevertheless, is quite rare. Guo et al. [68] reported in 2018 that LIN28A was capable of facilitating skin fibroblast proliferation and extracellular matrix synthesis so as to accelerate wound healing post-thermal injury, which could be suppressed by binding to miR-29a. It was recently reported in 2019 that lincRNA TINCR bound with SND1 to upregulate TGF-β1, which promoted escalated skin fibroblast proliferation and inflammation post burn [69].

**Figure 9:**
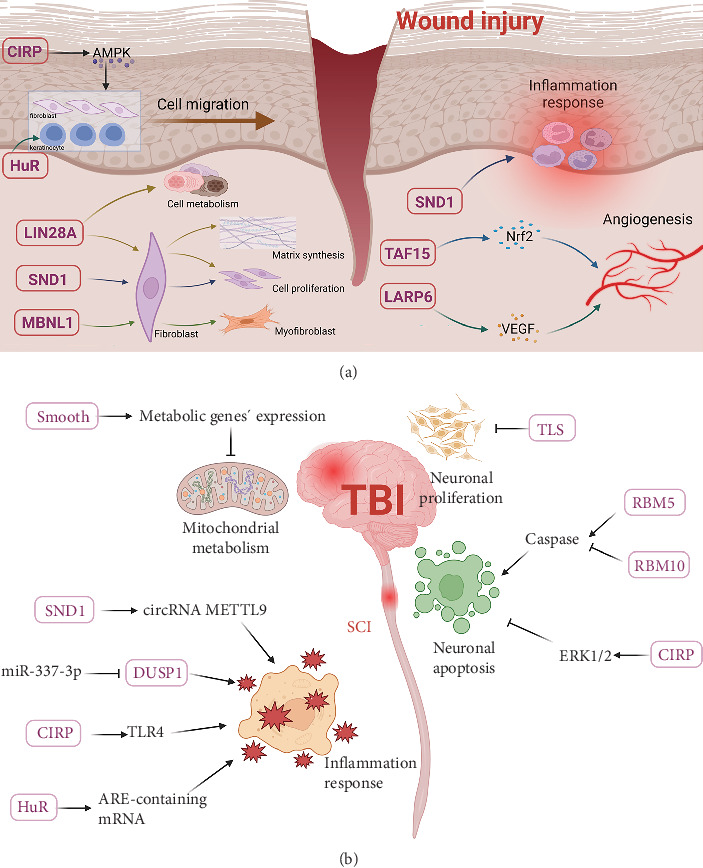
Schematic diagram. (A) RNA-binding proteins (RBPs) in the pathophysiological processes of cutaneous wound healing. (B) RBPs in the pathophysiological mechanisms of various traumatic injuries. Created in BioRender.

All in all, RBPs exert an essential function in the modulation of gene expression, RNA metabolism, and signaling pathways in the pathophysiological processes of cutaneous wound healing (Figure 9A).

### 5.2. RBPs in the Pathophysiological Mechanisms of Various Traumatic Injuries

#### 5.2.1. Cluster 3 (Blue) Was Characterized by the Prominent Phrases “RNA-Binding Protein,” “Traumatic Brain-Injury,” and “In-Vivo”

There are a variety of different causes of trauma, and TBI is responsible for the majority of disability and death of patients in traumatic injuries [6, 7]. TBI is an acquired injury to the brain caused by an external mechanical force that could lead to temporary or permanent damage, which constitutes a major source of morbidity and mortality all over the world [6, 7, 70]. In 1981, the first TBI rat model was characterized by a method of producing graded and reproducible focal cortical contusions [71]. After that, a lateral fluid percussion (LFP) and a controlled cortical impact (CCI) model of TBI in the rat were, respectively, described in 1989 and 1991, which opened the door to a whole new range of trauma research in TBI [72, 73]. Notably in 2001, utilizing quantitative real-time PCR (qRT-PCR) and the 2(-delta delta C (T)) method to process relative gene expression data was proposed by Livak et al. [74], which provided researchers with a new practical experimental tool.

Fused in sarcoma/translocated in liposarcoma (FUS/TLS) was early identified in 2009 to be engaged in the inhibition of neural progenitor self-replication in the hippocampus [75]. The activation of caspase was revealed by Jackson et al. [25] in 2015 to be stimulated by nuclear splicing factor RNA binding motif (RBM) 5 while inhibited by RBM10 in neurons after TBI. Afterward in 2022, RBM5 was further demonstrated to play a role in central nervous system by modulating cell signaling in a sex-dimorphic manner after TBI, but its inhibition did not affect hippocampal survival [76]. Moreover, CIRP expression was proposed by Kaneko [77] to be upregulated by mild hypothermia in mouse brains. Then Wang et al. [78] further indicate CIRP to suppress apoptosis of neurons by influencing the activation of extracellular signal-regulated kinase-1/kinase-2, thus presenting a neuroprotective effect during mild hypothermia following TBI. Additionally, with the help of RNA sequencing technology, smooth, an hnRNP-L homolog, was characterized to attenuate mitochondrial metabolism via post-transcriptional regulation of metabolic genes like isocitrate dehydrogenase in the subacute phase of TBI [27]. In addition, staphylococcal nuclease and tudor domain containing 1 (SND1) was shown to complex with circRNA METTL9 in astrocytes, attributing to neuron inflammation after TBI [79]. More recently, the oxidative stress and inflammatory response following TBI was displayed to be inhibited by regulation of short nucleolar RNA host gene 1 (SNHG1) on interaction between miR-377-3p and RBP dual-specific phosphatase-1 (DUSP1) [80]. Last but not the least, m (5)C RNA methyltransferase NOP2/Sun domain family member (NSUN) 6/7 were discovered to differentially expressed in Alzheimer's disease and TBI, which might be involved with the post-transcriptional regulation mechanisms in them [32].

#### 5.2.2. Cluster 4 (Red) Was Characterized by the Prominent Phrases “Messenger-RNA,” “Activation,” “Gene-Expression,” and “Protein”

Additionally in traumatic SCI, RBP Hu antigen R (HuR), ELAV (embryonic lethal, abnormal vision, Drosophila)-like 1 was early discovered to bind AU-rich elements (AREs) and stabilize ARE-containing mRNA in 2001 [81], which was then revealed to be activated in astrocytes and facilitate the inflammatory response in SCI [82]. Another ELAV family RBP, Hu antigen D (HuD), also known as ELAV (embryonic lethal, abnormal vision, Drosophila)-like 4, a developmentally regulated neuronal-specific protein, was firstly found to stabilize GAP-43 and facilitate the growth and differentiation of neurons [83]. Moreover, in optic nerve trauma, RBM3 expression was elevated in the eyes after hypothermia treatment, which might participate in some beneficial effects on the retina [84]. What is more, CIRP was also identified to exert effects on triggering mitochondrial DNA fragmentation and macrophage cell death via TLR4 signaling after traumatic injury [26]. Furthermore, the pathological alterations in FUS/TLS were also ulteriorly investigated to cause abnormal RNA metabolism, which might be linked with the degeneration of motor neurons following trauma [85]. More intriguingly, RBP fragile X-related protein 1 (FXR1) was indicated to interact with miR-132 to regulate post-traumatic stress disorder (PTSD)-like behaviors after some traumatic mental stress [86].

Taken together, RBPs greatly participate in the regulation of gene expression, RNA metabolism, and signaling pathways in the pathophysiological mechanisms of various traumatic injuries (Figure 9B).

## 6. Recommendations for Future Work

Through an analysis of high-frequency keywords, we elucidated the primary research domains within the RBP field and their association with trauma and burns. This analysis was complemented by a thorough review of pertinent research papers to discern key directions and hotspots in this domain. Employing key co-occurrence analysis, we identified two primary focuses: the involvement of RBPs in cell proliferation and migration and the activation of RBPs coupled with protein–protein interactions. These insights aim to guide forthcoming research endeavors and the clinical application of RBPs in the context of trauma and burns.

The current body of knowledge pertaining to RBPs in trauma and burns is in a continuous state of expansion, particularly in the fields of post-trauma inflammatory responses and the mechanisms underlying trauma and burns wound healing. This expansion is propelled by the ongoing development of high-throughput structural analysis methods, including but not limited to RIP-seq, ChIP-seq, RNP immunoprecipitation-microarray (RIP-Chip), ultraviolet light cross-linking and immunoprecipitation (CLIP), single-cell RNA-seq, and spatial RNA-seq [13, 87, 88]. These advanced methodologies contribute to the dynamic nature of research in this area.

## 7. Limitation

Nevertheless, there are still some limitations to our study. First, in order to ensure comprehensive citation data for analysis, we exclusively sourced papers from the WoSCC database. However, this approach may result in an insufficient compilation of all pertinent publications in the field of trauma and burn research on RBPs. Second, we only obtained publications before May 19, 2024, while the database is frequently updated, which would lead to the omission of some latest publications. Third, due to the predominant focus of RBP papers on mechanism studies in the field of trauma and burn, we encountered a scarcity of literature that hindered our ability to carry out a meta-analysis. Furthermore, RBPs encompass a broad category of proteins, each with distinct roles. Thus, the prior meta-analysis of RBP in TBI exhibited significant heterogeneity. However, when examining several RBPs in the Discussion, it becomes evident that RBPs have a significant influence on the prognosis of TBI. Fourth, due to the limitation of the inner logic of WoSCC retrieval system, no matter how we adjust the retrieval strategy, we could only find a balance between accuracy and completeness, leading to some irrelevant publications obtained while some relevant missed. However, we made our best to manually read as many articles as we could to attenuate the influences of the limitations. Despite these limitations, the present study still manages to provide a comprehensive review of the global status, developing trends and hotspots of RBPs in trauma and burn research.

## 8. Conclusion

In the present study, we obtained deep insight into the knowledge structures, global status, developing trends, and hotspots of research investigating RBPs in trauma and burns by bibliometrics analysis. The summarized two hotspots were RBPs in the pathophysiological mechanisms of various traumatic injuries and RBPs in the pathophysiological processes of cutaneous wound healing. This research field is rapidly developing, and hopefully our study will provide a valuable reference for scientific researchers and clinical practitioners.

## Figures and Tables

**Table 1:** Top 10 most productive countries/regions for RBPs in trauma and burn research.

Rank	Country/region	Publications (%)	SCP	MCP (%)
1	China	321 (59.6%)	299	22(6.9%)
2	United States	108 (20.0%)	83	25(23.1%)
3	Germany	25 (4.6%)	20	5 (20.00%)
4	Japan	15 (2.8%)	15	0 (0.00%)
5	United Kingdom	12 (2.2%)	6	6 (50.0%)
6	South Korea	9 (1.7%)	2	2 (22.2%)
7	Italy	5 (0.9%)	5	0 (0.00%)
8	Canada	4 (0.7%)	3	1 (25.00%)
9	India	4 (0.7%)	3	1 (25.00%)
10	Spain	4 (0.7%)	4	0 (0.00%)

Abbreviations: MCP, multiple-country publications; RBPs, RNA-binding proteins; SCP, single-country publications.

**Table 2:** Baseline characteristics of the included studies.

Population characteristics across the included studies	
Total number of studies	5
Total number of participants	5976

Data source and country	3 level I trauma centers for the TRACK-TBI Pilot Study/United States
	18 US level I trauma centers for the TRACK-TBI Pilot Study/United States
	22 ALERT-TBI trial investigational sites globally (15 in the United States and 7 in Europe)
	CENTER-TBI core study (version 3.0)/Europe and Israel

**Study characteristic**	

Data collection date range	2010–2018
Gender	Mixed sample

Abbreviations: ALERT-TBI, absence of intracranial injuries on a head CT in traumatic brain injury; CENTER-TBI, Collaborative European NeuroTrauma Effectiveness Research in Traumatic Brain Injury; TRACK-TBI, Transforming Research and Clinical Knowledge in Traumatic Brain Injury.

**Table 3:** Example with QUADAS-2 judgments to the left and QUADAS-C judgments to the right.

Study	Test	Risk of bias (QUADAS-2)				Applicability concerns (QUADAS-2)			Risk of bias (QUADAS-C)			
		P	I	R	FT	P	I	R	P	I	R	FT
Russell (2017)	CT	✓	✓	✓	✓	✓	✓	✓	✓	?	?	✓
	RBP	✓	✗	✓	✗	✓	✓	?				

Frederick (2022)	CT	✓	✓	✓	✓	✓	?	✓	✓	?	✓	✗
	RBP	✓	✓	✓	✗	✓	✓	✓				

Jeffrey (2018)	CT	✓	✓	✓	✓	✓	?	✓	✓	?	✓	✗
	RBP	✓	✓	✓	✗	✓	?	✓				

Isabel (2022)	CT	✓	✓	✓	✓	✓	✓	✓	✓	✓	✓	?
	RBP	✓	✓	✓	?	✓	✓	✓				

*Note:* Risk of bias is judged as “low,” ‘“high,” or “unclear.” If all domains (patient selection, index test, reference test, and flow and timing) are rated as “low,” then the risk of bias can be judged “low.” If any domain is rated as “high,” this flags the potential for bias. Concerns regarding applicability are rated in a similar way as the risk of bias: “low,” “high,” or “unclear” but apply only to three domains (patient selection, index test, and reference test). QUADAS-C is an extension to QUADAS-2 to assess the risk of bias in comparative diagnostic accuracy studies which compare two or more index tests in one study. Risk of bias is again judged as “low,” “high,” or “unclear.” ✓ indicates low risk; ✗ indicates high risk; and ? indicates unclear risk.

Abbreviations: CT, computed tomography; FT, flow and timing; I, index test; P, patient selection; R, reference standard; RBP, RNA-binding proteins.

## Data Availability

The datasets generated and/or analyzed during the current study are available on the Web of Science (WOS).

## Supporting Information

Supporting Information Figure S1: Author analysis. (A) Top 10 most local cited authors for RBPs in trauma and burn research. (B) Top 10 authors with most impact measured by *H*-index for RBPs in trauma and burn research. (C) Top 10 most productive authors' production over time for RBPs in trauma and burn research. Figure S2: Journal analysis. (A) Top 10 most local cited journals for RBPs in trauma and burn research. (B) Top 10 journals with most impact measured by *H*-index for RBPs in trauma and burn research. Figure S3: Publications and reference analyses. (A) Top 10 most local cited documents for RBPs in trauma and burn research. (B) Reference publication year spectroscopy for RBPs in trauma and burn research. Black line refers to the number of cited references, while red line refers to deviation from the 5-year median. (C) Historical direct citation network for RBPs in trauma and burn research. Figure S4: Altmetric analysis of “Lin28 enhanced tissue repair by reprogramming cellular metabolism”. (A) The AAS, ranking, and number of this research output. (B) The X demographics data shown were collected from the profiles of 77 users who shared this research output. (C) The Mendeley reader data shown were compiled from readership statistics for 666 readers of this research output. Figure S5: Keyword analysis. (A) Word cloud showing the top 50 most frequent words for RBPs in trauma and burn research. (B) Tree map demonstrating the top 50 most frequent words for RBPs in trauma and burn research.
