# Supplementary material for: Exploring the Research Focus of RNA-Binding Proteins in Trauma and Burns
Source: Anal Cell Pathol (Amst). 2024 Dec 30;2024:5587781. doi: 10.1155/ancp/5587781 (PMC11703594; doi:10.1155/ancp/5587781)
Supplement: Supporting Information — Figure S1: Author analysis. (A) Top 10 most local cited authors for RBPs in trauma and burn research. (B) Top 10 authors with most impact measured by H-index for RBPs in trauma and burn research. (C) Top 10 most productive authors' production over time for RBPs in trauma and burn research. Figure S2: Journal analysis. (A) Top 10 most local cited journals for RBPs in trauma and burn research. (B) Top 10 journals with most impact measured by H-index for RBPs in trauma and burn research. Figure S3: Publications and reference analyses. (A) Top 10 most local cited documents for RBPs in trauma and burn research. (B) Reference publication year spectroscopy for RBPs in trauma and burn research. Black line refers to the number of cited references, while red line refers to deviation from the 5-year median. (C) Historical direct citation network for RBPs in trauma and burn research. Figure S4: Altmetric analysis of “Lin28 enhanced tissue repair by reprogramming cellular metabolism”. (A) The AAS, ranking, and number of this research output. (B) The X demographics data shown were collected from the profiles of 77 users who shared this research output. (C) The Mendeley reader data shown were compiled from readership statistics for 666 readers of this research output. Figure S5: Keyword analysis. (A) Word cloud showing the top 50 most frequent words for RBPs in trauma and burn research. (B) Tree map demonstrating the top 50 most frequent words for RBPs in trauma and burn research. [file 5587781.f1.docx]

**Supplementary Materials**


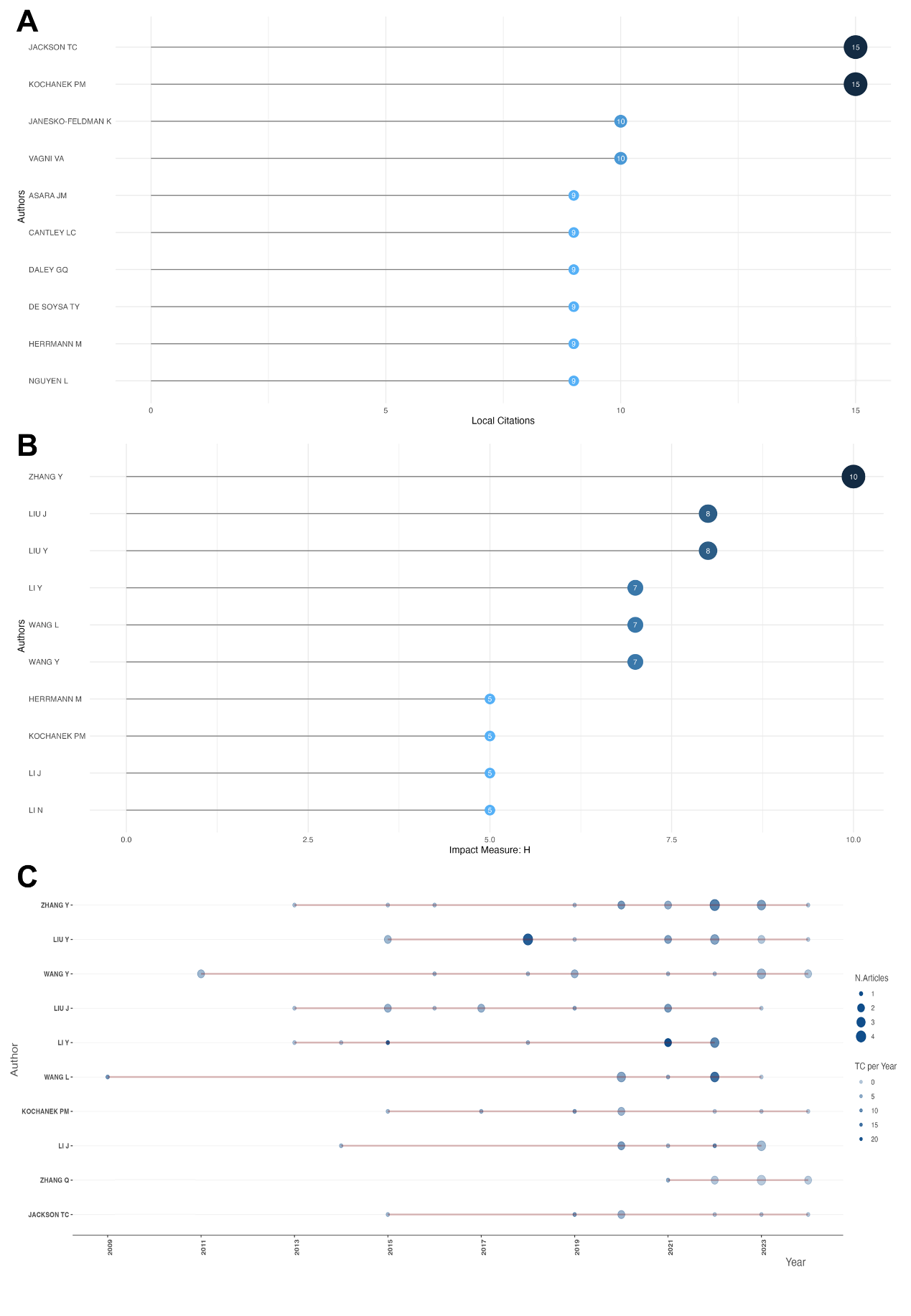


**Figure S1.** Author analysis.

(A) Top 10 most local cited authors for RBPs in trauma and burns research.

(B) Top 10 authors with most impact measured by H index for RBPs in trauma and burns research.

(C) Top 10 most productive authors’ production over time for RBPs in trauma and burns research.


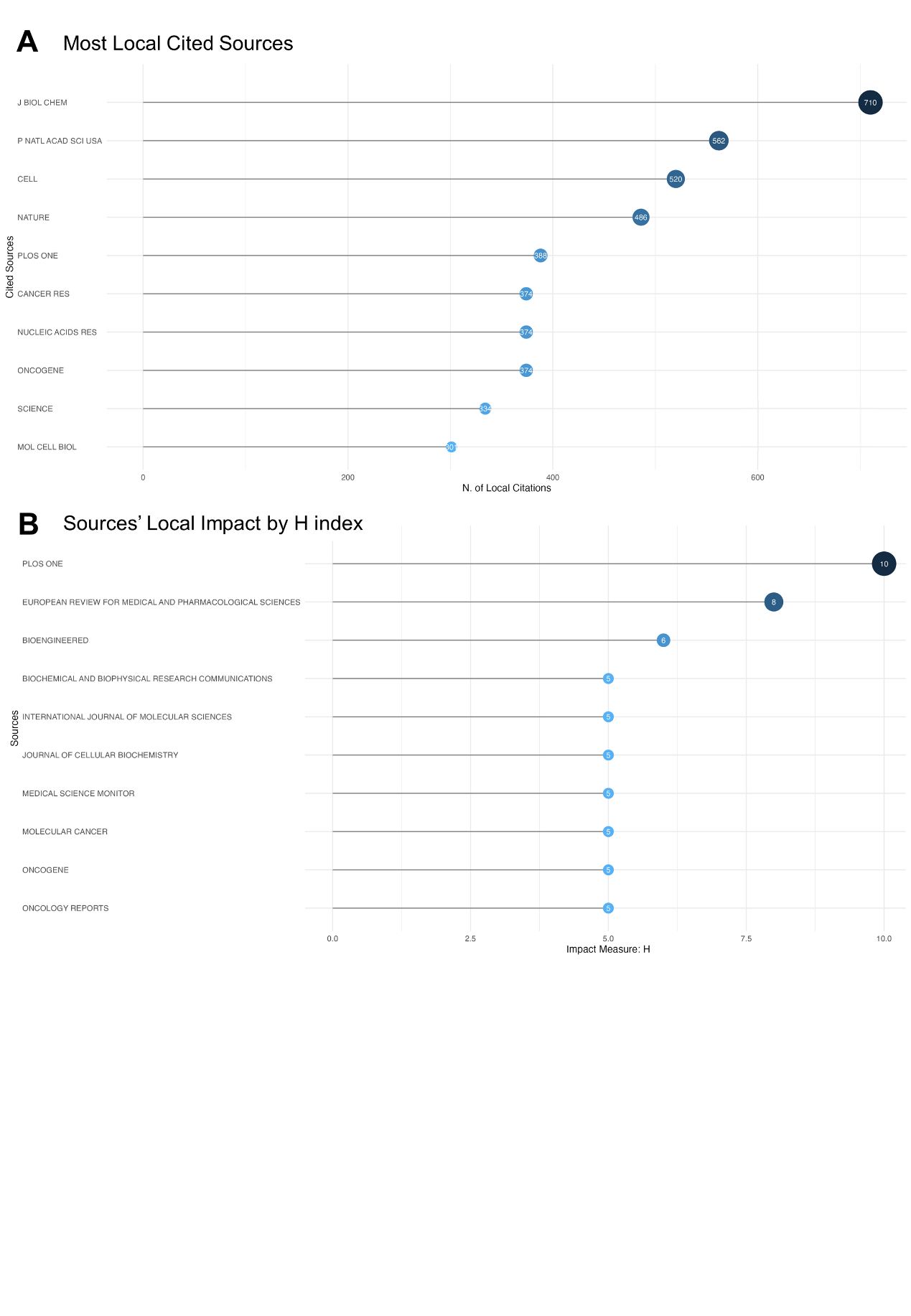

**Figure S2.** Journal analysis.

(A) Top 10 most local cited journals for RBPs in trauma and burns research.

(B) Top 10 journals with most impact measured by H index for RBPs in trauma and burns research.


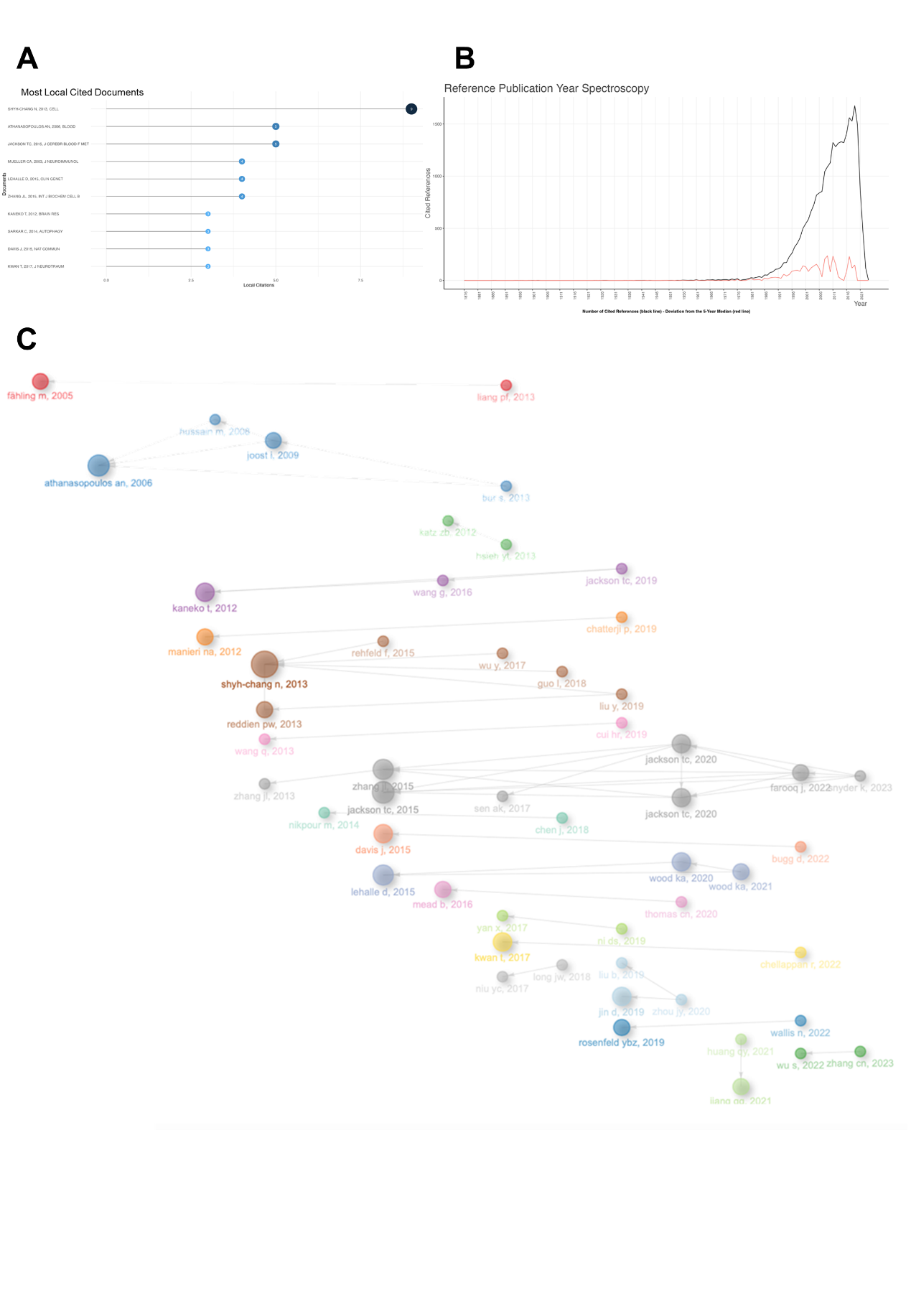


**Figure S3.** Publications and references analyses.

(A) Top 10 most local cited documents for RBPs in trauma and burns research.

(B) References publication year spectroscopy for RBPs in trauma and burns research. Black line refers to the number of cited references, while red line refers to deviation from the 5-year median. (C) Historical direct citation network for RBPs in trauma and burns research.


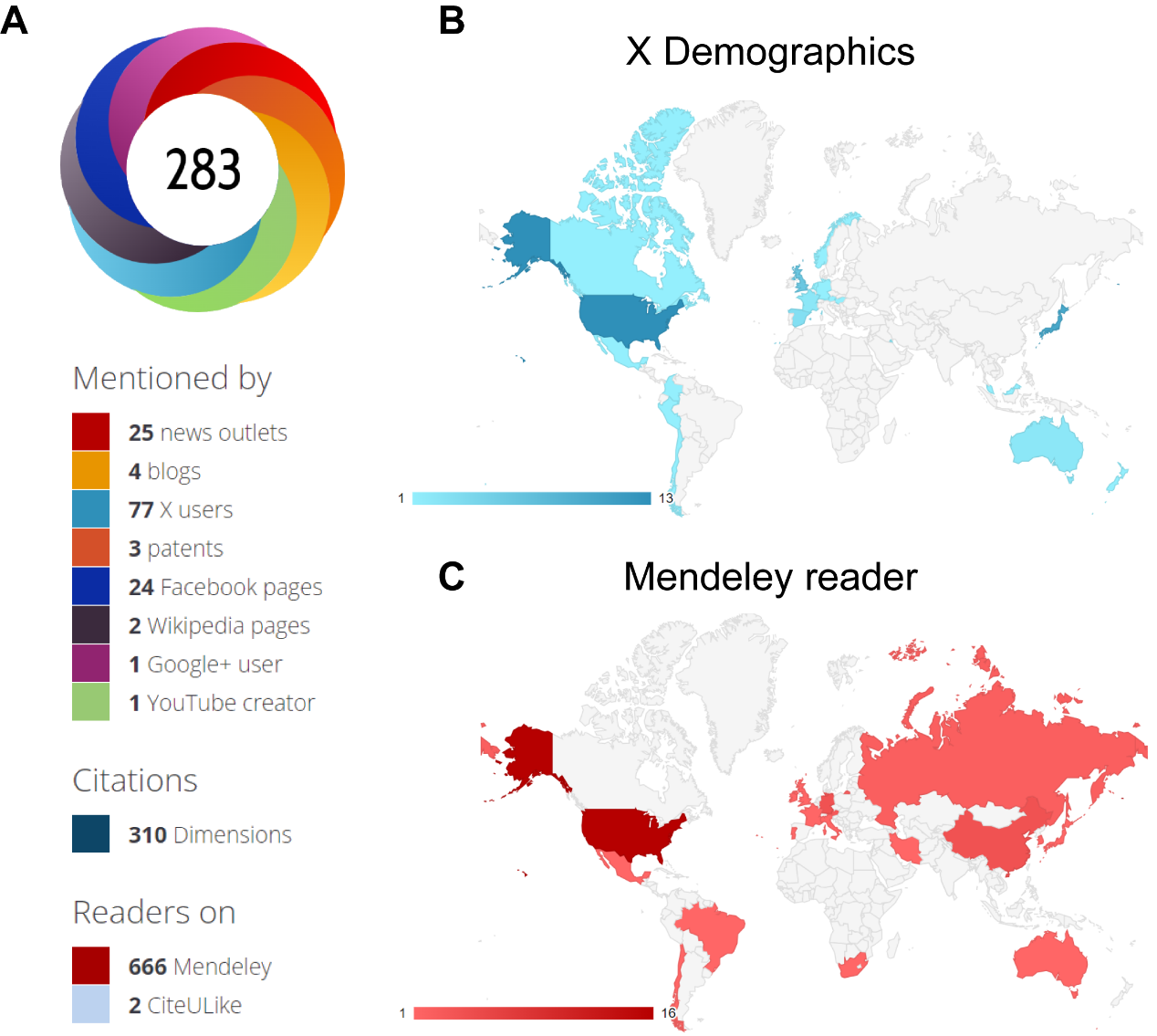


**Figure S4.** Altmetric analysis of “Lin28 enhanced Tissue Repair by Reprogramming Cellular Metabolism”.

(A) The AAS, ranking and number of this research output.

(B) The X demographics data shown were collected from the profiles of 77 users who shared this research output.

(C) The Mendeley readers data shown were compiled from readership statistics for 666 readers of this research output.


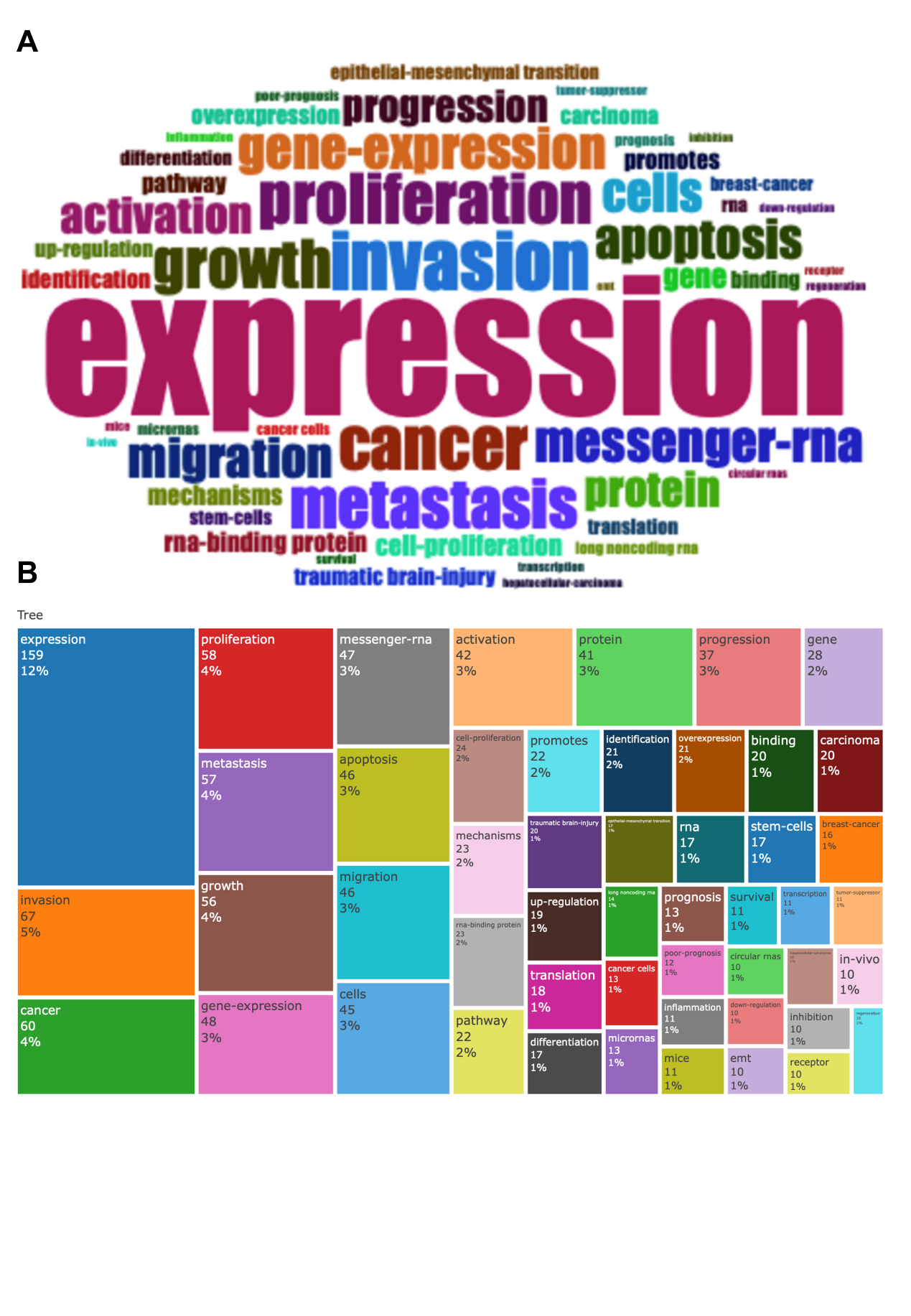


**Figure S5.** Keywords analysis.

(A) Word cloud showing the top 50 most frequent words for RBPs in trauma and burns research.

(B) Tree map demonstrating the top 50 most frequent words for RBPs in trauma and burns research.

**Supplementary Document 1**
